# A Preclinical Investigation of GBM-N019 as a Potential Inhibitor of Glioblastoma via Exosomal mTOR/CDK6/STAT3 Signaling

**DOI:** 10.3390/cells10092391

**Published:** 2021-09-11

**Authors:** Alexander T. H. Wu, Hsu-Shan Huang, Ya-Ting Wen, Bashir Lawal, Ntlotlang Mokgautsi, Thanh-Tuan Huynh, Michael Hsiao, Li Wei

**Affiliations:** 1 International Ph.D. Program for Translational Science, College of Medical Science and Technology, Taipei Medical University, Taipei 11031, Taiwan; chaw1211@tmu.edu.tw; 2 The PhD Program of Translational Medicine, College of Medical Science and Technology, Taipei Medical University, Taipei 11031, Taiwan; 3 Clinical Research Center, Taipei Medical University Hospital, Taipei Medical University, Taipei 11031, Taiwan; 4 Graduate Institute of Medical Sciences, National Defense Medical Center, Taipei 11490, Taiwan; huanghs99@tmu.edu.tw; 5 Taipei Heart Institute (THI), Taipei Medical University, Taipei 11031, Taiwan; 6 PhD Program for Cancer Molecular Biology and Drug Discovery, College of Medical Science and Technology, Taipei Medical University and Academia Sinica, Taipei 11031, Taiwan; bashirlawal12@gmail.com (B.L.); d621108006@tmu.edu.tw (N.M.); 7 Graduate Institute of Cancer Biology and Drug Discovery, College of Medical Science and Technology, Taipei Medical University, Taipei 11031, Taiwan; 8 School of Pharmacy, National Defense Medical Center, Taipei 11490, Taiwan; 9 PhD Program in Drug Discovery and Development Industry, College of Pharmacy, Taipei Medical University, Taipei 11031, Taiwan; 10 Department of Neurosurgery, Taipei Medical University-Wan Fang Hospital, Taipei 11031, Taiwan; 98142@w.tmu.edu.tw; 11 Taipei Neuroscience Institute, Taipei Medical University, Taipei 11031, Taiwan; 12 Center for Training and Scientific Research, Tam Anh General Hospital, Ho Chi Minh City 700000, Vietnam; tuanht@hcm.tahospital.vn; 13 Genomics Research Center, Academia Sinica, Taipei 11529, Taiwan; mhsiao@gate.sinica.edu.tw; 14 Department of Biochemistry, Kaohsiung Medical University, Kaohsiung 807378, Taiwan; 15 Graduate Institute of Injury Prevention and Control, College of Public Health, Taipei Medical University, Taipei 11031, Taiwan

**Keywords:** GBM-N019, exosome, glioma stem cell, palbociclib, drug resistance, combination therapy

## Abstract

Glioblastoma (GBM) is one of the most aggressive brain malignancies with high incidences of developing treatment resistance, resulting in poor prognoses. Glioma stem cell (GSC)-derived exosomes are important players that contribute to GBM tumorigenesis and aggressive properties. Herein, we investigated the inhibitory roles of GBM-N019, a novel small molecule on the transfer of aggressive and invasive properties through the delivery of oncogene-loaded exosomes from GSCs to naïve and non-GSCs. Our results indicated that GBM-N019 significantly downregulated the expressions of the mammalian target of rapamycin (mTOR), signal transducer and activator of transcription 3 (STAT3), and cyclin-dependent kinase 6 (CDK6) signaling networks with concomitant inhibitory activities against viability, clonogenicity, and migratory abilities of U251 and U87MG cells. Treatments with GBM-N019 halted the exosomal transfer of protein kinase B (Akt), mTOR, p-mTOR, and Ras-related protein RAB27A to the naïve U251 and U87MG cells, and rescued the cells from invasive and stemness properties that were associated with activation of these oncogenes. GBM-N019 also synergized with and enhanced the anti-GBM activities of palbociclib in vitro and in vivo. In conclusion, our results suggested that GBM-N019 possesses good translational relevance as a potential anti-glioblastoma drug candidate worthy of consideration for clinical trials against recurrent glioblastomas.

## 1. Introduction

Despite advancements in the knowledge and understanding of the mechanisms involved in the tumor biology of glioblastoma (GBM) and various treatment modalities over the last few decades, GBM remains one of the deadliest and the most common primary brain tumors, with tremendously poor prognoses [1]. The success of clinical trials of new chemotherapies and standard therapies has been disappointing [2] due to several factors including the drug delivery limiting features of blood–brain barrier (BBB), GBM immune-suppressive microenvironments, and structural fragility of the brain [3]. Furthermore, the existence of glioma-initiating cells (GICs) a type of glioma stem cell (GSC), mediates treatment failure, tumor recurrence, and invasive phenotypes of GBM [4]. As a carrier of oncogenes/proteins and other genetic information, exosomes are involved in the conversion of non-GSCs to GSCs and participate in stabilizing the GSC phenotypic integrity [5]. GBM is also characterized by complex and heterogeneous genotypes which limit the efficacy of drugs that target specific oncogenic signaling axes [6]. Thus, developing a multi-oncotarget treatment modality that targets GSCs and exosomes may improve the devastating prognoses of GBM.

The phosphatidylinositol-4,5-biphosphate 3-kinase (PI3K)/Akt/mammalian target of rapamycin (mTOR) signaling pathway has emerged as one of the most deregulated oncogenic pathways that contribute to aggressive phenotypes, drug resistance, and poor prognoses in GBM patients [7]. However, due to several limiting factors [8,9,10,11], efforts to target this signaling axis failed to improve the prognoses of GBM patients [8]. Importantly, the inability of mTOR inhibitors to target mTORC2 epitomizes another major clinical limitation of targeted therapy [12]. Therefore, targeting mTORC2 would overcome the limitations of mTORC1 inhibitors and provide a sound therapeutic strategy for GBM. This is supported by preclinical evidence of the critical roles of mTORC2 in GBM biology and the extenuating effect of targeting mTORC2 on GBM growth, invasive phenotypes, and drug resistance [13,14,15], thus paving the way for personalized and targeted therapy. 

Cyclin-dependent kinases (CDKs) are members of the serine/threonine protein kinase family that regulates cell division and transcription [16,17]. Unrestricted cell cycle progression and high cellular growth due to aberrant CDK4/6 signaling have been identified as hallmarks of astrocytic tumorigenesis and glioma progression in most GBM cases [18]. Hyper-expression of CDK4/6 was also documented in several other cancer types [19,20,21,22,23]. Although mono-therapeutic inhibitors that target CDK4/6 signaling pathways have been developed [24,25], their efficacy in GBM remains disappointing [18], thus accentuating the need for synergistic contributions from other agents.

Summing up the above literature with the clinical data from The Cancer Genome Atlas (TCGA) database strongly suggests that CDK6/mTOR/STAT3 overexpression is correlated with a high glioma grade, lower survival, and poor prognosis in glioblastoma patients [18,23]. The presence of GSCs and oncogene delivery features of exosomes [5], together with aberrations of CDK6/mTOR/STAT3 oncogenic pathways, concomitantly contribute to aggressive phenotypes and the failure of therapeutic strategies against GBM [25]. New therapeutic strategies are urgently needed to address all the challenges mentioned above to improve patients’ survival. 

Palbociclib is an oral selective inhibitor of CDK4/6, which leads to phosphorylation of RB1 and cell-cycle arrest [26]. RB1 status, therefore, becomes a determinant of tumor sensitivity to palbociclib therapy. Disappointedly, about 11% of GBM show complete loss of RB1 transcript expression [27], rendering them resistant to palbociclib [28]. Clinical studies have also demonstrated that CDK4/6 inhibitor alone showed sub-optimal efficacy for recurrent glioblastoma [29,30,31]. Thus, in combination with other therapies, palbociclib has been vigorously tested and proven effective in some patients [28,31,32,33,34,35,36]. Specifically, mTOR inhibitor with palbociclib showed increased efficacy against GBM [37,38]. In addition, there are several ongoing trials testing combinations of palbociclib with immunotherapy, including avelumab and pembrolizumab (NCT02778685; NCT02779751; and NCT03147287) [39]. Collectively, these findings strongly suggested that targeting mTOR/CDK6 associated signaling is a potential new target for developing GBM therapeutics. 

Anthraquinone-derived heterocyclic scaffolds have been explored for drug design, discovery, and development [40], and drug candidates from this class have demonstrated antitumor activities in various studies [41,42,43,44,45]. GBM-N019 is a novel member of a series of anthraquinone-derived, tetraheterocylic azathioxanthone derivatives. Herein, we demonstrated for the first time through a series of in vitro and in vivo studies that GBM-N019 significantly compromised the viability and tumorigenic features of GBM cells via downregulation of STAT3, Akt, mTOR, nuclear factor (NF)-κB, and CDK6 signaling networks. We also found that GBM-N019 halted the exosomal cargo delivery of Akt, mTOR, p-mTOR, and RAB27A, and attenuated the tumorsphere-derived exosomes (exosphere; Exosp) mediated drug resistance and aggressive phenotypes of GBM. In addition, we demonstrated that GBM-N019 synergized with palbociclib to achieve more positive treatment outcomes. Interestingly GBM-N019 suppressed not only mTORC1 but also mTORC2, thus overcoming the challenges of previous therapies and renewed our hope for success against GBM. 

## 2. Materials and Methods

### 2.1. Mining and Analysis of Glioblastoma Clinical Data 

Differential gene expression profiles of the mTOR, STAT3 and CDK6 signature across The Cancer Genome Atlas (TCGA) databases were analyzed using the Gene Expression Profiling Interactive Analysis (GEPIA) server (http://gepia.cancer-pku.cn/(accessed on 26 June 2021) [46]. Tumor IMmune Estimation Resource (TIMER2.0) resource (http://timer.cistrome.org/ (accessed on 28 June 2021)) was used to analyze the expression correction of mTOR, STAT3, and CDK6 [47]. The Kaplan–Meier survival plot was used to assess the overall survival (OS) of the cohort. We also explored the cancer genomic dataset using the cBioPortal tool (http://www.cbioportal.org/ (accessed on 27 June 2021)) to analyze genomic alterations of mTOR, STAT3 and CDK6 and its associated prognostic relevance in 585 glioblastoma patients in TCGA, PanCancer Atlas [48,49]. Protein–protein interactions (PPIs) and functional enrichment analysis including Kyoto Encyclopedia of Genes and Genomes (KEGG) pathways and Gene Ontology (GO) enriched in mTOR, STAT3 and CDK6 signature were conducted using the Search Tool for Retrieval of Interacting Genes (STRING, vers. 10.5, (https://www.string-db.org/ (accessed on 27 June 2021)) with the adjusted threshold confidence set to 0.900 [50] and Enrich (https://maayanlab.cloud/Enrichr/enrich# (accessed on 28 June 2021)) [51,52]. 

### 2.2. Target Identification and Molecular Docking of GBM-N019 

We used the SwissADME tools, a web tool for predicting the macromolecular targets of a bioactive small molecule, to identify the potential bio-targets of GBM-N019. Swiss Target Prediction operates on the "principle of similarity" which states that the two most similar molecules are prone to have similar properties and bio-targets [53]. We used the blood–brain barrier (BBB) Prediction Server (https://www.cbligand.org/BBB/ (accessed on 9 July 2021)) which operates based on the support vector machine (SVM) and LiCABEDS algorithms to analyze the BBB permeation ability of GBM-N019 [54]. Molecular docking was performed using AutoDock Vina (vers. 0.8, the Scripps Research Institute, La Jolla, CA, USA) [55] with all parameters set to default values, and all bonds in the ligand rotated freely while considering the receptor to be rigid. A grid box of 40 × 40 × 40 Å at X, Y, and Z dimensions and a spacing of 1.0 angstrom were used [55]. The crystal structures of the targets, i.e., CDK6 (PDB ID: 1JOW), mTOR (PDB ID: 5FLC), and STAT3 (PDB ID: 4ZIA), were retrieved from the Protein Data Bank in the PDB file format and subsequently converted to the pdbqt format. The chemical structure of the ligand (GBM-N019) was prepared using Avogadro molecular builder and visualization tool vers. 1.XX (http://avogadro.cc/ (accessed on 11 July 2021)) [56], while that of the clinical CDK inhibitor (palbociclib) was retrieved in mol file format from the PubChem database. These two ligands were converted into the PDB format using the PyMOL Molecular Graphics System, vers. 1.2r3pre and subsequently converted into the pdbqt format using Auto Dock Tools 1.5.6. The ligands were prepared for molecular docking by deletion of H_2_O molecules, adjustment of polar hydrogen, and addition of Kollman charges [57]. The docked ligand–receptor complex was visualized and analyzed using the Discovery studio visualizer vers. 19.1.0.18287 (BIOVIA, San Diego, CA, USA) [58].

### 2.3. Cell Lines and Culture

The human GBM cell lines (U251 and U87MG) were obtained from American Type Culture Collection (ATCC, Manassas, VA, USA). Cells were cultured in fetal bovine serum (FBS; 10%) and penicillin/streptomycin (1%) supplemented with RPMI-1640, and incubated in humidified 5% CO_2_ at 37 °C. Culture media were replaced after 3 days, and cells were subcultured at 90%~95% confluence.

### 2.4. Drugs and Chemicals 

**Figure 1:**
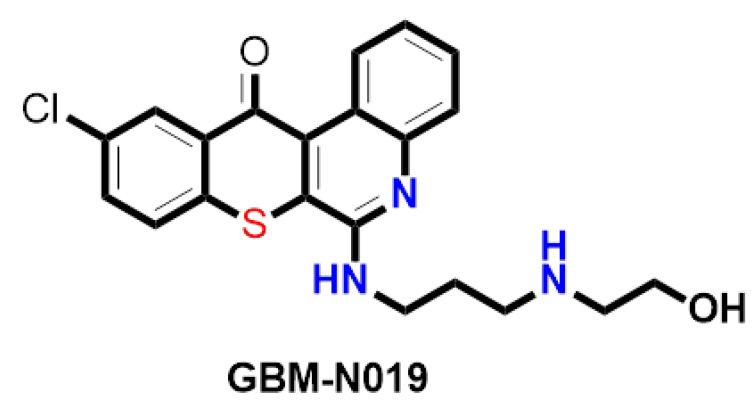
Chemical structure of GBM-N019.

Palbociclib was purchased from Selleckchem (Houston, TX, USA), while GBM-N019 was synthesized through established protocols in our lab [40]. The chemical structure of GBM-N019 is presented in Figure 1. A 10 mM stock solution of the drugs was prepared in dimethyl sulfoxide (DMSO) and kept frozen at a temperature of −20 °C. Gibco®RPMI 1640 Medium (Cat. No. 11875085) was obtained from Thermo Fisher Scientific, Inc. (Waltham, MA, USA). Other reagents and chemicals, including sulforhodamine B (SRB), DMSO, FBS, phosphate-buffered saline (PBS), TRIS base, and acetic acid, were procured from Sigma Aldrich (St. Louis, MO, USA).

### 2.5. Cytotoxicity Assay

Cytotoxicity assays were conducted using established protocols of the SRB reagent [59]. Approximately 4 × 10^3^ of U251 or U87MG cells were seeded in each well of 96-well plates overnight (15 h). After incubation, cells were treated with various doses of drugs (palbociclib and/or GBM-N019) for 2 days and washed thrice with PBS (1%) followed by sequential treatment with trichloroacetic acid (10%), double-distilled (dd)H_2_O, and 0.4% SRB, and then incubated for 1 h. Acetic acid (1%) was used to remove any unbound free SRB dye. The plate was air-dried and re-dissolved in 20 mmol/L Tris-base, and the absorbance was read at a wavelength of 570 nm. The nature of the interaction (additive, synergistic, or antagonistic) between GBM-N019 and palbociclib when used in combined treatment against GBM cells was assessed using the Chou–Talalay algorithm-based isobologram method [60]. The percentage cell viability following treatment with palbociclib: GBM-N019 at different ratios was recorded and computed with CompuSyn software (ComboSyn, Paramus, NJ, USA). Accordingly, CIs of ˂1, =1, and ˃1 were, respectively, interpreted as synergistic, additive, and antagonistic.

### 2.6. Wound-Healing and Colony-Formation Assays

In total, 3 × 10^5^ of U251 and U87MG cells were seeded in each well of a silicon-based two-well bilayer culture insert, carefully placed in a sterile culture dish, and incubated at 37 °C in a humidified 5% CO_2_ atmosphere for 24 h. The culture insert was carefully removed, and cells were treated with GBM-N019. The migration of cells across the bilayer was monitored and photographed under a microscope (10×) and quantified with Image J software (https://imagej.nih.gov/ij/ (accessed on 9 July 2021)). The colony forming abilities of parental and Exo^sp^-co-cultured U87MG and U251 cells were determined by seeding 500 cells into each well of six-well plates (Corning, Taipei, Taiwan) overnight and treating them with different concentrations of GBM-N019. The cells were incubated for a week. Subsequently, cells were fixed and stained with SRB reagent, and the colonies were counted.

### 2.7. Tumorsphere Formation and Isolation of Tumorsphere-Derived Exosomes

The tumorsphere-formation assay was conducted according to established protocols [61]. In total, 1 × 10^4^ U87MG and U251 cells were seeded into each well of six-well ultra-low-adhesion plates (Corning, Corning, NY, USA) and cultured in serum-free media (SCM020, Merck, Darmstadt, Germany), supplemented with 20 ng/mL human epidermal growth factor (hEGF), 20 ng/mL human basic fibroblast growth factor (hbFGF), 0.2% heparin, and 1% antibiotics. The cell culture was allowed to grow for 10 days, and independent aggregated cells (tumorspheres) with a diameter of ≥ 100 µm were counted with an inverted phase-contrast microscope and harvested for further analysis. To isolate tumorsphere-derived exosomes (Exo^sp^), U87MG and U251 tumorsphere culture media were mixed with a total exosome extraction kit (Thermo Fisher Scientific, Taipei, Taiwan) at a ratio of 10:1 and processed following the manufacturer’s protocol. Cluster of differentiation 9 (CD9) and Alix were used as exosomal markers. The particle sizes of the exosomes were determined using the qNano (IZON Science) and results were visualized using the Izon Control Suite V3.2.2.268 (IZON Science)

### 2.8. Co-Cultured Assays

To further extend the effect of exosomes cross-talk, we tested whether exosome could transfer oncogenic, chemoresistance, and aggressive phenotypes to the naïve GBM cells. The isolated exosomes (Exo^sp^) isolated from tumorspheres of U251 or U87MG cell lines were co-cultured with corresponding parental U251 and U87MG cells. The U251 or U87MG cells (5 × 10^5^ cells/well) were seeded in six-well plates, and 100 µL (1 mg) of the respective exosomes were added and cultured for 48 h. The exosome co-cultured cells (^Exosp^U251 and ^Exosp^U87MG cells) were subsequently harvested for further analyses.

### 2.9. RNA Isolation and Real-Time Polymerase Chain Reaction (PCR)

Total RNA from GBM cells was isolated and purified following the TRIzol-based protocol (Life Technologies). Five hundred nanograms (500 ng) of total RNA was reverse-transcribed (RT) using a Qiagen OneStep RT-PCR Kit (Qiagen, New Taipei City, Taiwan), and the PCR was performed using a Rotor-Gene SYBR Green PCR Kit (400, Qiagen). Primers used for the quantitative (q)PCR experiments are listed in Supplementary Data (Table S1).

### 2.10. Western Blot Analysis

An established protocol for the Western blot analysis [62] was used to analyze and compare protein expression profiles between different treatment groups. Following the various indicated drug treatments, total protein lysates from cells were separated by 10% sodium dodecylsulfate polyacrylamide gel electrophoresis (SDS-PAGE), transferred onto polyvinylidene difluoride membranes (Bio-Rad Laboratories, Hercules, CA, USA), and blocked (with 5% skimmed milk) for non-specific binding. Finally, the membrane was immunoblotted with primary antibodies (Supplementary Table S2) for 15 h, and secondary antibodies (horseradish peroxidase-linked anti-rabbit and mouse, 1:5000) for 2 h. Protein signals from the membrane were detected with an enhanced chemiluminescence kit (Thermo Fisher Scientific, Waltham, MA, USA).

### 2.11. In Vivo Studies

Female NOD/SCID mice (6 weeks old with an average weight of 25.42 ± 1.52 g) were purchased from BioLASCO (Taipei, Taiwan) and maintained under the SPF conditions at the Animal Center of Taipei Medical University. All experiments involving animals were conducted in compliance with the Affidavit of Approval of animal use protocol (approval no. LAC-2017-0161) issued by Taipei Medical University. Mice were randomly placed into four groups (n = 5) and intracranially injected with 5 × 10^5^ U87MG dissociated tumorspheres as described previously [63]. Four groups were established: GBM-N019 only (5 mg/kg five times/week I.P), palbociclib only (150 mg/kg five times/week, P.O), the combination of GBM-N019 and palbociclib, and the control receiving a sham injection. The body weights were monitored weekly using an electronic weighing balance while the tumor growth was monitored using IVIS 200 bioluminescence imaging system (Caliper Life Science Inc., Hopkinton, MA, USA) every week [64]. The change in tumor burden was determined by total photon flux (photons/s), which was calculated as flux per unit area and unit angle over the region of interest (ROI). At the end of the study, surviving mice were humanly euthanized as described previously [65,66], after which tumor samples were harvested and processed for further analyses.

### 2.12. Statistical Analysis

Experiments were conducted in triplicates, and data were analyzed using the GraphPad Prism version 6.04 for Windows, GraphPad Software, (La Jolla California USA). Results are presented as the mean ± standard deviation (SD). Data from treatment groups were compared with the control using Student’s t-test. Statistical significance was considered at *p* < 0.05 (*), *p* < 0.001 (**), and *p* < 0.0001 (***).

## 3. Results

### 3.1. mTOR, STAT3, and CDK6 Are Key Oncogenic Signatures of Disease Progression, Therapy Failure, and Poor Prognosis in GBM Patients

**Figure 2:**
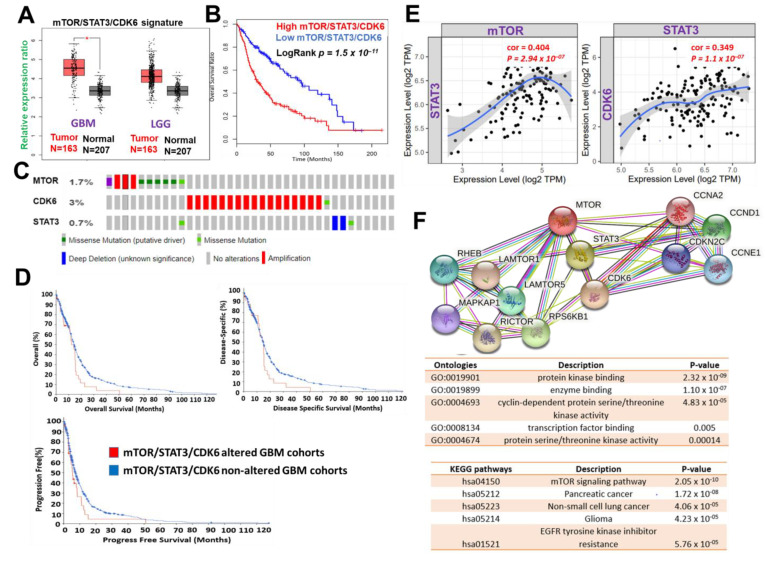
mTOR, STAT3 and CDK6 is an important oncogenic signature of disease progression, therapy failure, and poor prognosis in GBM patients. (**A**) Cumulative mTOR, STAT3, and CDK6 expression levels in the patients with GBM and low-grade glioma (LGG) from TCGA and GTEx datasets. (**B**) Kaplan–Meier plots of the cumulative survival of GBM patients. (**C**) Frequency of genetic alterations of mTOR, STAT3 and CDK6 signature in glioblastoma (TCGA, PanCancer Atlas) dataset. (**D**) Kaplan–Meier plots of the overall survival, disease-specific, and disease progressive free survival of GBM cohorts with genetically altered mTOR, STAT3 and CDK6 signature. (**E**) Correlation analysis of the expression between STAT3 and mTOR (**left**) and between CDK6 and STAT3 (**right**) from GBM TCGA databases. Both *p*-values and Spearman’s rank correlation coefficient (cor) are indicated. (**F**) Protein–protein interaction networks of mTOR, STAT3 and CDK6 signature (upper panel) and the enriched KEGG pathways and gene ontologies in the mTOR, STAT3 and CDK6 clustering network.

The use of bioinformatics databases and software for biomarkers and drug target predictions have greatly enhanced the process of drug design and development [67]. Our analysis of clinical data from the TCGA database indicated that mTOR, STAT3, and CDK6 are collectively hyper-expressed in both low-grade glioma and GBM cohorts (Figure 2A). These hyper-expressions were also found to correspond with the poor overall survival of cancer patients (Figure 2B). We also mined the clinical data of glioblastoma cohorts in TCGA, PanCancer Atlas and found that alterations in mTOR, STAT3, and CDK6 constituted 5% of the genetic alteration occurring in GBM patients (Figure 2C). These genetic alterations were found to be associated with shorter overall survival, disease-specific survival, and progression-free survival of the cohorts (Figure 2D). Additionally, we identified a strong correlation between expressions of mTOR and STAT3 (*P* = 2.95 × 10 ^−7^, r = 0.405) and STAT3, as well as between STAT3 and CDK6 (*P* = 1.1 × 10 ^−7^, r = 0.349) (Figure 2E). Analysis of functional protein association networks of mTOR, STAT3, and CDK6 identified (PPI enrichment p-value: 4.17 × 10^−8^) CCNA2, CCNE1, CCND3, CCND1, Rictor, Raptor, MLST8, RHEB, and FKBP1A to be functional partners associated with their expressions and oncogenic properties in GBM (Figure 2F). The PPI networks of mTOR, STAT3, and CDK6 were enriched in cancer-associated KEGG pathways and ontologies, among which cyclin-dependent protein serine/threonine kinase activity, transcription factor binding, mTOR signaling pathway, pancreatic cancer, non-small cell lung cancer, glioma, EGFR tyrosine kinase inhibitor resistance were those enriched (Figure 2F lower panel). Collectively, this preliminary computational study revealed that mTOR, STAT3, and CDK6 is an important oncogenic signature that is associated with disease progression, therapy failure, and poor prognosis of GBM cohorts

### 3.2. mTOR, STAT3, and CDK6 Are Druggable Targets for a Novel Drug-like Multitarget Small Molecule (GBM-N019)

**Figure 3:**
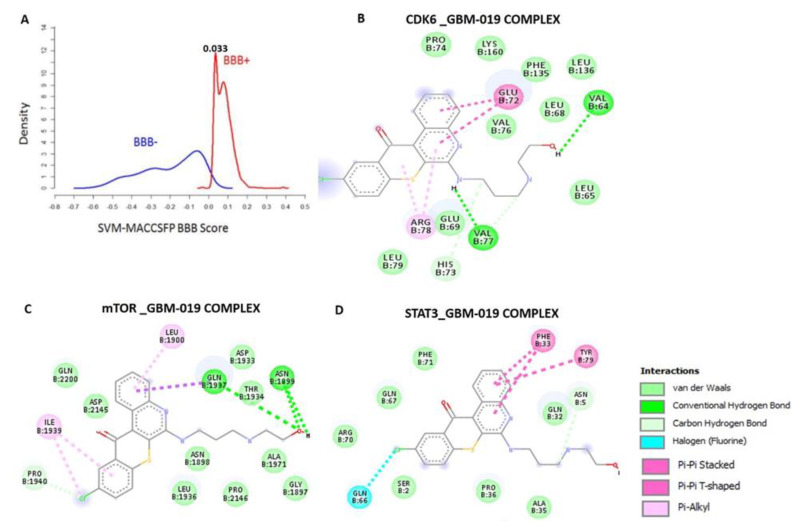
Blood–brain barrier permeation and molecular docking profiles of GBM-0N19 with STAT3, mTOR, and CDK6. (**A**) BBB permeation curve of GBM-N019 as measured by the support vector machine (SVM) and LiCABEDS algorithms of BBB prediction server. The two-dimensional (2D) representations of the ligand–receptor complexes, showing the interacting amino acid residues and the type of interactions occurring between the GBM-N019 and (**B**) CDK6 (**C**) mTOR and (**D**) STAT3.

Having established the oncogenic roles of mTOR, STAT3 and CDK6 signatures in GBM, we evaluated the drug-likeness and therapeutic properties of GBM-N019. Our computational study revealed that the GBM-N019 is BBB permeant (Figure 3A), it has acceptable absorption, distribution, metabolism, and excretion (ADME) characteristics and met all criteria of Lipinski’s rule of five (Supplementary Table S3). Furthermore, with computer-based drug target predictions and using GBM-N019 as a query molecule, we identified a number of targetable proteins, most of which were previously implicated in the poor prognosis of GBM. Among these predicted target proteins, enzymes (33.3%), kinases (26.7%), proteases (13.3%), electrochemical transporters (13.3%), and family A G protein-coupled receptors (13.3%) were the most repartitioned targeted classes (Supplementary Figure S1). Specifically, three members of cyclin-dependent kinases (CDK4, -6, and -9) and three members of the PI3K family (PI3K p110-delta subunit (PIK3CD), PI3K p110-beta subunit (PIK3CB), and PI3K p110-gamma subunit (PIK3CG)) were predicted to be targeted by GBM-N019. Additionally, using GBM-N019 as the query molecules, mTOR, NF-κB, and STAT6 appeared as part of the topmost targets (Supplementary Table S4). Molecular docking studies revealed that GBM-N019 docked well into the binding cavity of (Figure 3B), mTOR (Figure 3C), and STAT3 (Figure 3D) with binding affinities of −7.6, −8.1, and −6.9 Kcal/mol, respectively. The ligand receptors complex was bound by several hydrogen bonding, alkyl interactions. Furthermore, the complex was stabilized by various hydrophobic contacts and several Van der Waal forces surrounding the ligand backbone (Figure 2B; Supplementary Table S5). Interestingly, GBM-N019 bind to CDK6 with the same affinity that palbociclib has for CDK6 (ΔG = −8.1 Kcal/mol); however, the affinities of GBM-N019 for mTOR and STAT3 are lower than that of their respective standard inhibitors; Dactolisib (ΔG = −9.2 Kcal/mol vs. −7.6 Kcal/mol) and SH-4-54 (ΔG = −7.3 Kcal/mol vs. −6.9 Kcal/mol) (Supplementary Figure S2). Collectively, our computational study revealed that GBM-N019 is a novel drug candidate as an inhibitor of mTOR/STAT3/CDK6 signature.

### 3.3. GBM-N019 Curbed the Viability and Tumorigenic Features of GBM Cells via Downregulation of NF-κB/Akt/mTOR, STAT3, and CDK6 Signaling In Vitro

**Figure 4:**
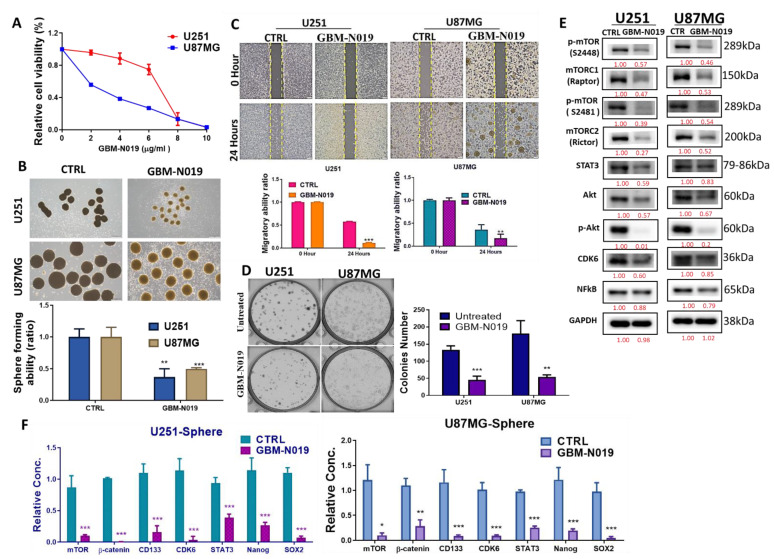
GBM-N019 treatment inhibited tumorigenic properties via downregulating mTOR, STAT3, and CDK6 signaling pathways in vitro (**A**) GBM-N019 treatment significantly hampered the viability of U251 and U87MG cells in dose-dependent manners. GBM-N019 treatment significantly reduced the tumorsphere forming abilities (**B**), migratory ability (**C**), and colony forming (**D**) abilities of both U251 and U87MG cells. (**E**) Western blots of GBM-N019-treated U251 and U87MG cells exhibited lower expression levels of -mTOR, mTORC1, mTORC2, STAT3, Akt, p-Akt, CDK6, and NF-κB. (**F**) qPCR analysis showed decreased gene expression levels of mTOR, β-catenin, CD133, CDK6, STAT3, Nanog, and SOX2 in both U251 and U87MG cells after treatment with GBM-N019. * *p* < 0.05, ** *p* < 0.01, *** *p* < 0.001.

Following our in silico predictions, we evaluated the anti-GBM effects of GBM-N019 using human GBM U251 and U87MG cell lines. We demonstrated that GBM-N019 significantly reduced the viability of U251 and U87MG cells in a dose-dependent manner (Figure 4A). Treatment with GBM-N019 also significantly compromised the sphere forming ability (Figure 4B), migratory capacity (Figure 4C), and clonogenicity (Figure 4D) of U251 and U87MG cells. Furthermore, the expression of p-mTOR, mTORC1, mTORC2, STAT3, Akt, p-Akt, CDK6, and NF-κB, in U251 and U87MG cells were significantly downregulated by GBM-N019 treatment (Figure 4E). Our qPCR analysis revealed that the inhibition of the sphere forming ability of U251 and U87MG cells by GBM-N019 was concomitantly associated with decreased expressions of mTOR, β-catenin, CD133, CDK6, STAT3, Nanog, and SOX2 (Figure 4F). Altogether, these findings suggest that GBM-N019 suppressed tumorigenic features of GBM cells via downregulation of NF-κB/Akt/mTOR, STAT3, and CDK6 signaling axis.

### 3.4. Tumorsphere-Derived Exosomal Cargo of Oncogenes Mediated Treatment Resistance and Aggressive Phenotypes of GBM

**Figure 5:**
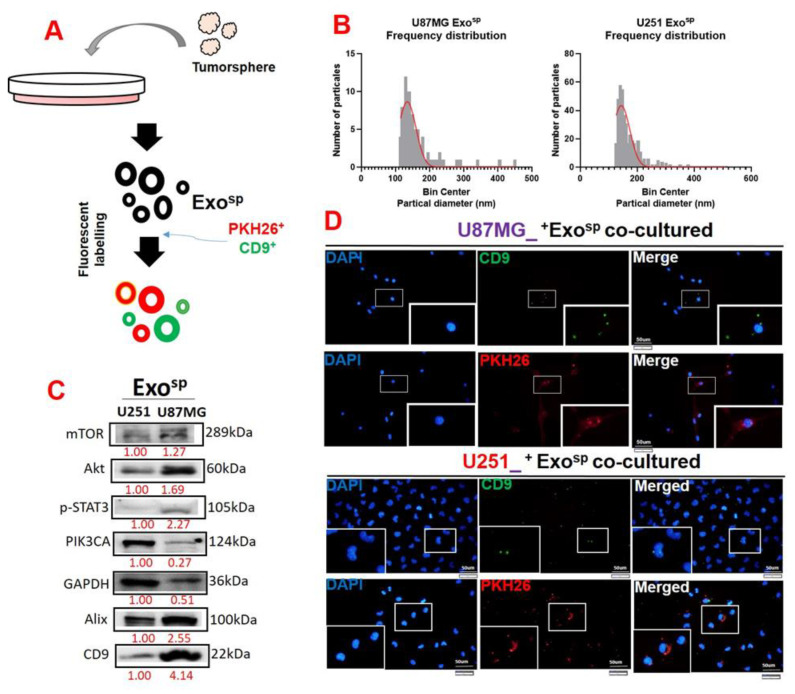
Characterization of tumorsphere-derived exosomes (Exo^sp^). (**A**) Schematic representation of exosome isolation from the U87MG and U251 tumorsphere. (**B**) Particle analysis demonstrated that exosomes (Exo^sp^) isolated from U87MG and U251 tumorspheres were in the 130 and 170 nm diameter range. (**C**) Western blots of isolated Exo^sp^ from U87MG and U251 cells indicated the exosomal cargo of p-STAT3, Akt, mTOR, and PIK3CA. (**D**) Immunofluorescence imaging depicting the uptake of Exo^sp^ in the recipient cells; CD9 (green fluorescence) and PKH26 (red fluorescence) were both exosomal trackers. Numbers in red indicate the relative expression ratio.

**Figure 6:**
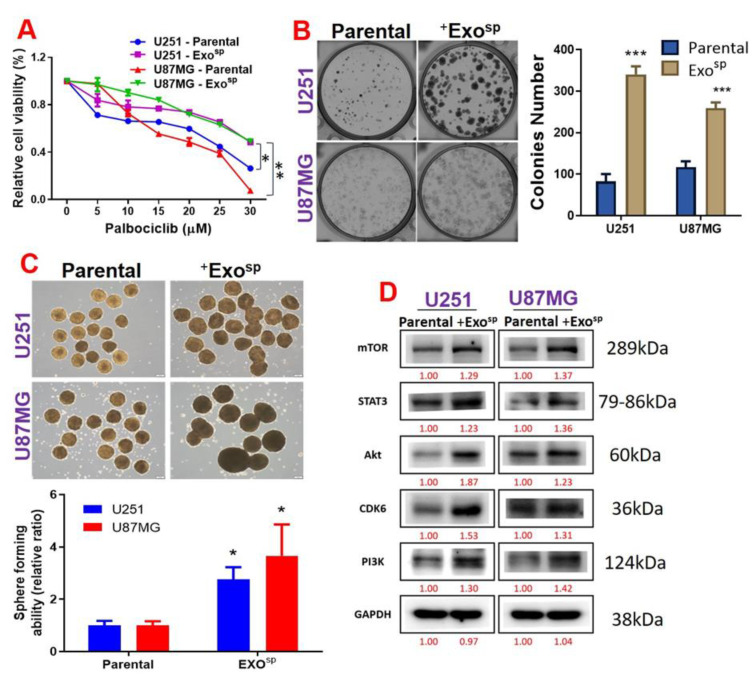
Tumorsphere-derived exosomes (Exo^sp^) promoted the expression of mTOR, STAT3 and CDK6 expressions, palbociclib resistance, and tumorigenic properties of GBM cell lines. (**A**) Cell viability curve showing that U87MG and U251 cells co-cultured with Exo^sp^ exhibited low sensitivity to palbociclib treatments compared to their parental counterparts. U87MG and U251 cells co-cultured with Exo^sp^ also exhibited higher (**B**) colony forming ability, (**C**) sphere generating, and (**D**) expression levels of mTOR, STAT3, CDK6, Akt, and PI3K compared to their parental counterparts. Numbers in red indicate the relative expression ratio. * *p* < 0.05, ** *p* < 0.01, *** *p* < 0.001.

To investigate the oncogenic properties of exosomes, we isolated exosomes from sphere populations of U251 and U87MG cells and cultured parental cells with their respective exosomes. We found that the isolated exosome (Exo^sp^) from the U87MG and U251 sphere produced major peaks at 130 and 170 nm in diameter, respectively (Figure 5A,B). In addition, the isolated exosome (Exo^sp^) contained Akt, mTOR, PIK3CA, and activated STAT3 (p-STAT 3) (Figure 5C). Our immunofluorescence imaging of the U87MG and U251 co-cultured with the isolated Exo^sp^ demonstrated that the CD9/PHK26 fluorescent-labeled Exo^sp^ were uptaken by the recipient U251 and U87MG cells (Figure 5D). Furthermore, U251 and U87MG cells cultured with the Exo^sp^ (^Exosp^U251 and ^Exosp^U87MG cells) were less sensitive to palbociclib treatment (Figure 6A) and exhibited higher colony forming (Figure 6B) and sphere generating (Figure 6C) abilities than their respective parental counterparts. Accompanying these tumorigenic features were increased expressions of mTOR, PI3K, Akt, STAT 3, and CDK6 in ^Exosp^U251 and ^Exosp^U87MG cells compared to the parental cells (Figure 6D). 

### 3.5. GBM-N019 Synergized with Palbociclib and Re-Sensitized the Exo^sp^-Transformed GBM Cell to Palbociclib Treatment

**Figure 7:**
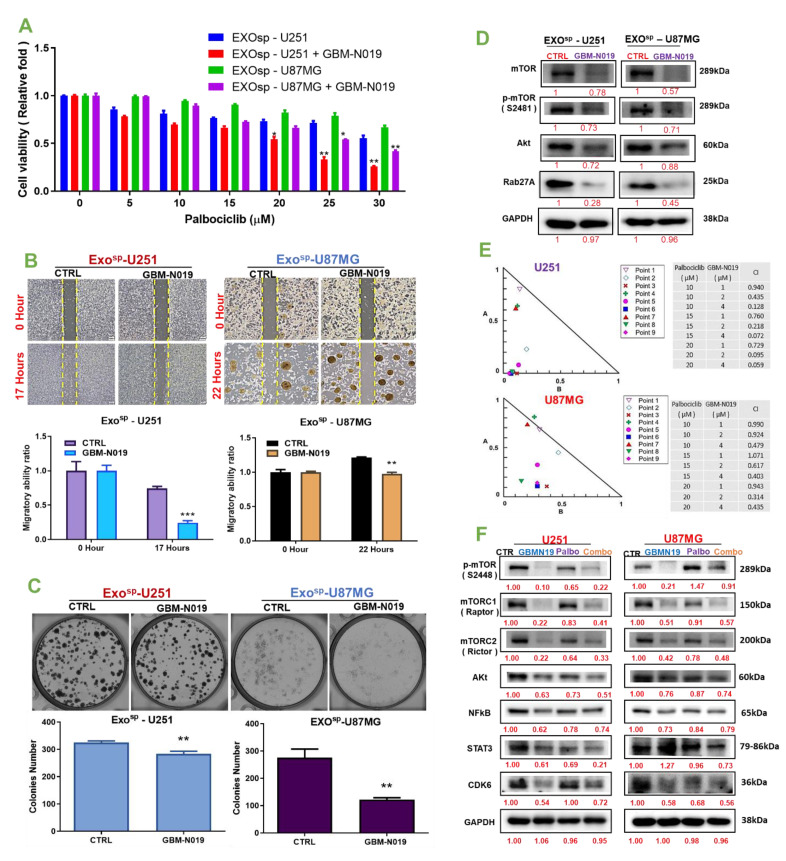
GBM-N019 synergized with palbociclib and re-sensitized the Exo^sp^-transformed GBM cells to palbociclib treatment. (**A**) Bar graph of cell viability showing that GBM-N019 pretreatment increased the sensitivity of Exo^sp^-transformed U251 and U87MG cells to palbociclib treatment. GBM-N019 treatment significantly reduced the (**B**) migratory and (**C**) colony forming ability, and (**D**) expression levels of mTOR, p-mTOR, Akt, and RAB27A in Exo^sp^-transformed U251 and U87MG cells. (**E**) Isobologram analysis showing that the synergistic effects of GBM-N019 and palbociclib was achieved in different concentration combinations in both U251 and U87MG cells. (**F**) Western blot showing that the expression levels of p-mTOR, mTORC1, mTORC2, Akt, NF-κB, STAT3, and CDK6 in U251 and U87MG cells following treatment with the combined therapy of palbociclib: GBM-N019 were more significantly reduced compared to their expression levels in cells treated with individual drugs. Numbers in red indicate the relative expression ratio. ** *p* < 0.01, *** *p* < 0.001.

The importance of combination therapy in alleviating the hurdle of drug resistance and improving treatment outcomes in cancer patients cannot be overemphasized [2,68]. Herein, we investigated the possibilities of enhancing the efficacy of a clinically available CDK4/CDK6 inhibitor, palbociclib, by combining it with our novel small molecule (GBM-N019). Firstly, we accessed the effect of GBM-N019 on exosome-mediated drug resistance and aggressive phenotypes of GBM and found that GBM-N019 re-sensitized ^Exosp^U251 and ^Exosp^U87MG cells to palbociclib treatment. Co-treatment with palbociclib and GBM-N019 enhanced the efficacy of palbociclib and resulted in lower cell viability in ^Exosp^U251 and ^Exosp^U87MG cells compared to cells treated with palbociclib alone (Figure 7A). In addition, we found that compared to the untreated counterpart, treatment with GBM-N019 significantly compromised the migratory (Figure 7B) and colony generating (Figure 7C) abilities of ^Exosp^U251 and ^Exosp^U87MG cells. Furthermore, our comparative Western blot analysis revealed lower expression levels of mTOR, p-mTOR, Akt, and RAB27A in GBM-N019 treated ^Exosp^U251 and ^Exosp^U87MG cells compared to their control counterparts (Figure 7D). In order to further establish the effect of GBM-N019 and palbociclib as a combined therapy in glioblastoma, we treated U251 and U87MG cells using different combination ratios of palbociclib: GBM-N019 and analyzed treatment responses with the aid of a Chou–Talalay-based algorithm, a popular software for drug combination analyses. Interestingly a synergistic effect (combination index (CI) of < 1) of GBM-N019 and palbociclib were achieved in different concentration combinations in both U251 and U87MG cells (Figure 7E). Furthermore, the expression levels of p-mTOR, mTORC1, mTORC2, Akt, NF-κB, STAT3, and CDK6 in U251 and U87MG cells following treatment with the combined therapy of palbociclib: GBM-N019 were more significantly reduced compared to their expression levels in cells treated with individual drugs (Figure 7F). Collectively, our findings indicate that GBM-N019 synergized with palbociclib and re-sensitized the exosomal-mediated drug resistance GBM to palbociclib treatment.

### 3.6. GBM-N019 Suppressed GBM Tumorigenesis and Enhanced the In Vivo Efficacy of Palbociclib

**Figure 8:**
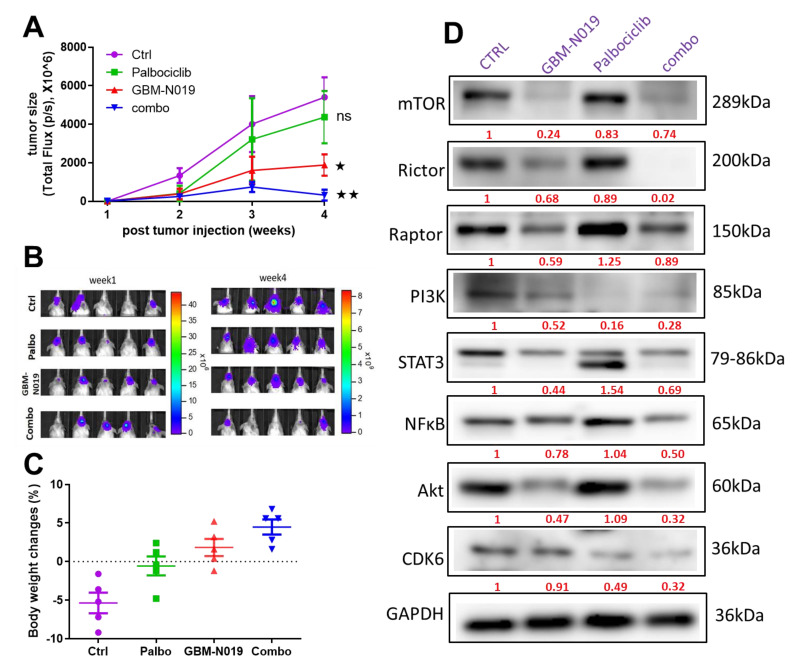
GBM-N019 suppressed U87MG tumorsphere-induced tumorigenesis and enhanced the in vivo efficacy of palbociclib. (**A**) An average tumor volume versus time curve, and (**B**) bioluminescence image shows that treatments with GBM-N019 and palbociclib significantly delayed U87MG tumor growth and burden than GBM-N019 treatment alone. Treatment with palbociclib had no significant therapeutic effect (*p* > 0.05) on the tumor progression when compared with the control. (**C**) Graph of percentage body weight changes of mice; GBM-N019 alone or in combination with palbociclib showed a normal body weight gain in the animals over the treatment course, suggesting no apparent systemic toxicity. (**D**) Western blots from tumor samples revealed decreased expressions of mTOR, mTORC1(Raptor), mTORC2(Rictor), PI3K, STAT3, NFkB, Akt, and CDK6 in mice treated with GBM-N019 alone or in combination with palbociclib. Numbers in red indicate the relative expression ratio. * *p* < 0.05, ** *p* < 0.01.

Following our in vitro studies, we utilized a mouse xenograft model to evaluate the in vivo tumor-suppressive effect of GBM-N019 and combination with palbociclib. GBM-N019 alone significantly reduced U87MG tumorsphere-induced growth (Figure 8A,B) and posed no apparent systemic toxicity, as reflected by the steady increase in average body weight in the animals (Figure 8C). Palbociclib alone did not show a significant inhibitory function on the tumor growth compared to GBM-N019 (*p* > 0.05). However, palbociclib treatment did appear to exhibit a slight inhibitory effect compared to the control (no statistical significance); mice receiving palbociclib also did not show systemic toxicity (Figure 8C). Interestingly, the combination of GBM-N019 and palbociclib demonstrated the highest degree of suppression on the U87MG tumorsphere-induced tumor growth (Figure 8A–C). Molecularly, GBM-N019 treatment and the combined regimen resulted in the decreased expressions of mTOR, mTORC1(Raptor), mTORC2 (Rictor), PI3K, STAT3, NFkB, Akt, and CDK6; while the combined treatment negatively impacted the expression level of these oncogenic markers to a greater extent (Figure 8D). These findings suggested that GBM-N019 might have sensitized U87MG tumorspheres towards palbociclib, leading to an increased efficacy. 

## 4. Discussion

The lack of effective therapeutics for managing aggressive phenotypes of GBM rationalized the need to identify therapeutic biomarkers and develop novel bioactive molecules with better efficacy and safety profiles than the current standard of care [4,5,6]. The present study provided evidence that PI3K/Akt/NF-κB/mTOR/STAT3/CDK6 signaling network collectively contributes to generating cancer stemness in GBM. We presented preclinical support of therapeutic intervention GBM-N019 against naïve and aggressive GBM. Our bioinformatics analyses initially suggested that three major oncogenic markers, mTOR, STAT3, and CDK6, are collectively upregulated in GBM and are strongly correlated with the poor survival of GBM patients. Consistent with our findings, the implicative role of PI3K/Akt/NF-κB/mTOR, STAT3, and CDK6 signaling pathways in the GBM phenotype, drug resistance, and poor prognoses has been well recognized in clinical and experimental studies [69,70]. Additionally, STAT3 and CDK6 have been associated with cancer-associated fibroblast and immune cell infiltration and are considered biomarkers of poor prognosis in multiple cancers [23]. Furthermore, our analysis of PPI clustering networks of mTOR, STAT3, and CDK6 identified some functional partners that are known regulators of GBM growth and invasive properties [71,72,73,74]. These PPI clustering networks were also found to be enriched in pathways and ontologies that are associated with tumor progression and drug resistance. Consequently, downregulation of the PI3K-Akt/NF-κB/mTOR, STAT3, and CDK6 signaling pathways was reportedly used to abrogate GBM oncogenic properties and re-sensitized aggressive glioblastomas to chemotherapy [75,76,77].

Non-covalent interactions, including the hydrogen bonds, hydrophobic contacts, and ionic interactions, play pivotal roles in stabilizing the interaction between small molecules and protein targets [78]. Our molecular docking analysis predicted that GBM-N019 interacts with STAT3, CDK6, and mTOR by several hydrogen bonding, alkyl interactions, hydrophobic contacts, and Van der Waal forces created on its backbone with respective amino acid residues of the receptor-binding cavity. Besides the non-covalent interactions, the Van der Waal forces would stabilize the complex [79]. Interestingly, our in silico model indicated that GBM-N019 binds CDK6 with the same affinity as palbociclib binds CDK6 (ΔG = −8.1 Kcal/mol). However, the affinities of GBM-N019 for mTOR and STAT3 were lower than that of their respective specific inhibitors, Dactolisib (ΔG = –9.2 Kcal/mol vs. –7.6 Kcal/mol) and SH-4-54 (ΔG = −7.3 Kcal/mol vs. −6.9 Kcal/mol). Altogether, our molecular docking experiments suggested that GBM-N019 has the molecular properties to interact efficiently with CDK6, STAT3, and mTOR and supported our in vitro and in vivo findings.

Exosomes are considered mediators of intercellular cell communications [80]. Our findings revealed that GSC-derived exosomes contained and transferred oncogenic molecules and chemoresistance phenotypes to the naïve cells. In line with our observations, a previous study reported that exosomes derived from 5-fluorouracil-resistant cells transferred p-STAT3 and conferred drug resistance to drug-sensitive cells [81]. Other studies demonstrated the extracellular vesicle/exosome-mediated metastatic properties of the bladder [64] and colorectal [82] cancers. Our findings partially elucidated the mechanism through which GSCs confer stemness and invasive properties to non-GSCs by cargo conveyer of oncogenic Akt, mTOR, PI3K, and activated STAT3 (p-STAT3). Therefore, identifying a therapeutic agent that can halt this stemness transfer process could be a salvaging strategy to curb the aggressiveness of GBM. 

Herein, we used multiple approaches to demonstrate the therapeutic properties of GBM-N019 in experimental glioblastomas. We found that GBM-N019 significantly inhibited the GBM phenotypes and expression of mTORC/AKT/STAT3/CDK6 signaling pathways. Monotherapies that target individual oncogenes of the above pathways failed to maintain durable clinical outcomes due to compensatory activations of other pathways [8,11]. It is, therefore, noteworthy that our novel small molecule, GBM-N019, single-handedly downregulated the PI3K/Akt/NF-κB/mTOR, STAT3, and CDK6 signaling pathways. Interestingly, GBM-N019 not only suppressed mTORC1 but also mTORC2, thus overcoming the challenge of previous therapy [12]. In addition, we established that GBM-N019 inhibited the exosomal uptake and transfer of oncogenic molecules, enhanced palbociclib’s sensitivity, and prevented the exosome-mediated gain of aggressive properties.

Increased cases of drug resistance prompted the idea of combination therapy in cancer treatment [2,68]. Accordingly, we observed that treatment of GBM cells with palbociclib alone decreased GBM cell viability in vitro and no significant activity in the in vivo model. In agreement with our findings, experimental and clinical limitation of palbociclib monotherapy against cancer, particularly glioblastoma has been reported in previous studies [30,83,84,85,86,87]. In addition, the loss of body weight in the palbociclib treated rats echoes the clinical reports on palbociclib [85,88]. However, the combination with an mTOR inhibitor has been suggested as a viable combination strategy for treating recurrent glioblastomas [30]. Therefore, by combining palbociclib with a small active molecule, it may be possible to decrease palbociclib’s therapeutic dose, which appears to be a promising way. Interestingly, we observed that GBM-N019 synergized with palbociclib and significantly delayed tumor growth with concomitant suppression of p-mTOR, mTORC1, mTORC2, Akt, NF-κB, STAT3, and CDK6 when compared to individual therapies. In line with our findings, previous studies have reported that inhibitors of CDK6 mediated mTOR activation [89] while combined therapy of an mTOR inhibitor and a CDK6 inhibitor synergized to achieve enhanced inhibition of tumor growth in glioblastoma [18], head and neck [90] and pancreatic [89] cancers. Furthermore, target inhibition of STAT3 has been reported to re-sensitize palbociclib-resistant ER-positive breast cancer to palbociclib treatment [91].

Consistent with our observation that mice treated with GBM-N019 alone or in combination with palbociclib exhibited no significant loss of body weight compared to the control, Chowdhary et al. [92] and Hans et al. [93] reported that no therapy associated toxicities were observed in cancer patients receiving palbociclib in combination with other therapies. Collectively, our results suggest that combining palbociclib with GBM-N019 may be a good combination strategy to combat GBM. One of the limitations of our study is that the immune-deficient mouse model used does not fully mimic the immunogenicity status of GBM patients, and thus this model cannot fully address the potential immune-modulating functions of GBM-N019. However, GBM-N019-targeted mTOR/CDK6/STAT3 signature, supported by a wealth of literature, contributes to tumor progressions, stemness, distant metastasis, and regulation of immune checkpoint blockade therapy outcome [23,57,74,87,91]. Thus, the suppressive effect of GBM-N019 on mTOR, CDK6, and STAT3 signaling pathways could be of translational relevance to the immunogenicity status of GBM patients, and this issue is currently under vigorous investigation.

On the general translational relevance, GBM-N019 exhibits favorable pharmacokinetic properties and meets the criteria for a drug candidate [94]. BBB penetration is an essential limiting factor in developing treatments for glioblastoma [95]. Interestingly, our in silico studies predict GBM-N019 to be a BBB permeant molecule and hence possess good translational relevance as a potential anti-glioblastoma drug candidate. However, our results are limited to a murine model, further studies are required to establish the clinical benefit of GBM-N019 in human GBM.

## 5. Conclusions

In conclusion, exosomes derived from GSCs mediated the transfer of stemness and invasive properties to U251 and U87MG cells. GBM-N019 efficiently impaired the exchange of oncogenic molecules and rescued cells from the exosomal-mediated gain of drug resistance, stemness, and aggressive properties. Furthermore, GBM-N019 was synergized to enhance the anti-GBM activities of palbociclib. These findings suggested that GBM-N019 possesses good translational relevance as a potential anti-glioblastoma drug candidate worthy of consideration for clinical trials against recurrent glioblastomas.

## Data Availability

The datasets generated and/or analyzed in this study are available on reasonable request.

## Supplementary Materials

The following are available online at https://www.mdpi.com/article/10.3390/cells10092391/s1, Table S1: Primers used for the qPCR experiments. Table S2: Primary antibodies used for Western blot analysis, Table S3: SWISSADME predicted GBM-N019 potential drug target, Table S4: Drug likeness and ADME property of GBM-N019, Table S5: Molecular docking profiles of GBM-0N19 with STAT3, mTOR and CDK6. Figure S1. SWISSADME predicted classes of GBM-N019 potential drug target. Figure S2: Molecular docking profiles of standard inhibitors with STAT3, mTOR and CDK6. Figure S3: Uncropped images of Western blots used in the figures.
