# Supplementary material for: A Preclinical Investigation of GBM-N019 as a Potential Inhibitor of Glioblastoma via Exosomal mTOR/CDK6/STAT3 Signaling
Source: Cells. 2021 Sep 11;10(9):2391. doi: 10.3390/cells10092391 (PMC8471927; doi:10.3390/cells10092391)
Supplement: Supplementary file 1 [file cells-10-02391-s001.zip › cells-1386872-supplementary.pdf]

Supplementary table S1: Primers used for the qPCR experiments.

	(5′→3′)
Actin -F	TTG TTA CAG GAA GTC CCT TGC C
Actin -R	ATG CTA TCA CCT CCC CTG TGT G
AKT1 -F2	CTA CCC ACA CAG CAG TAC GC
AKT1 -R2	AAG TCG CTG GTG TTA AGC CG
CD133 -F	TTC TTG ACC GAC TGA GAC CCA
CD133 -R	TCA TGT TCT CCA ACG CCT CTT
CDK6 -F	CCA GAT GGC TCT AAC CTC AGT
CDK6 -R	AAC TTC CAC GAA AAA GAG GCT T
β-catenin -F	ACT GGC AGC AAC AGT CTT ACC
β-catenin -R	TTT GAA GGC AGT CTG TCG TAA T
STAT3 -F	ACC AGC AGT ATA GCC GCT TC
STAT3 -R	GCC ACA ATC CGG GCA ATC T
mTOR -F	CTG GGA CTC AAA TGT GTG CAG TTC
mTOR -R	GAA CAA TAG GGT GAA TGA TCC GGG
Nanog -F	ATG GAG GAG GGA AGA GGA GA
Nanog -R	GAT TTG TGG GCC TGA AGA AA
SOX2 -F	ACA CCA ATC CCA TCC ACA CT
SOX2 -R	GCA AAC TTC CTG CAA AGC TC

Supplementary table S2: primary antibodies used for western blot analysis.

β -actin	1:5000	Proteintech, Beta Actin Antibody, Rabbit Polyclonal , 20536-1-AP
α-tubulin	1:2000-1:5000	Proteintech, alpha Tubulin Antibody, Rabbit Polyclonal , 11224-1-AP
Akt	1:1000	Cell Signaling, Akt Antibody, Rabbit, #9272
p-Akt	1:1000	Cell Signaling, Phospho-Akt (Ser473) (D9E) XP® Rabbit mAb #4060
CDK6	1:1000	Cell Signaling, CDK6 (DCS83) Mouse mAb, #3136
GAPDH	1:5000-20000	Proteintech, GAPDH Antibody, Rabbit Polyclonal, 10494-1-AP
mTOR	1:1000	Cell Signaling, mTOR (7C10) Rabbit mAb #2983
p-mTOR	1:1000	Cell Signaling, Phospho-mTOR (Ser2481) Antibody #2974
p-mTOR	1:500-1:1000	ABclonal, Phospho-mTOR-S2448, Rabbit mAb, AP0115
NF-κB	1:1000	Cell Signaling, NF-κB p65 (D14E12) XP® Rabbit mAb #8242
STAT3	1:1000	Cell Signaling, Stat3 (79D7) Rabbit mAb, #4904
p-STAT3	1:1000	Cell Signaling, Phospho-Stat3 (Tyr705) (D3A7) XP® Rabbit mAb, #9145
STAT3	1:500-1:2000	ABclonal, STAT3, Rabbit pAb, A11216
p-STAT3	1:500-1:2000	ABclonal, Phospho-Stat3-Y708, Rabbit pAb, AP0070
STK4	1:500-1:2000	ABclonal, STK4, Polyclonal Antibody, A8043
TGF-β 1	1:1000	Proteintech, TGF-Beta 1 Antibody, Rabbit Polyclonal, 18978-1-AP
WISP1	1:500-1000	Proteintech, WISP1 Antibody, Rabbit polyclonal , 18166-1-AP
KRAS	1:2000	Proteintech, KRAS Antibody, Rabbit polyclonal, 12063-1-AP
p-Raf 1 (Ser259)	1:1000	EnoGene, Raf-1 (Phospho-Ser259) Rabbit, E011006
p-Mek 1 (Ser221)	1:1000	EnoGene, MEK1 (Phospho-Ser221) Rabbit, E11-7147A
GAPDH	1:5000-10000	Proteintech, GAPDH Antibody, Rabbit Polyclonal, 10494-1-AP

Supplementary table S3: SWISSADME predicted GBM-N019 potential drug target

Target	Common name	Uniprot ID	ChEMBL ID	Target Class
**PI3-kinase p110-delta subunit**	PIK3CD	O00329	CHEMBL3130	**Enzyme**
**PI3-kinase p110-beta subunit**	PIK3CB	P42338	CHEMBL3145	**Enzyme**
**PI3-kinase p110- gamma subunit**	PIK3CG	P48736	CHEMBL3267	**Enzyme**
**Cyclin-dependent kinase 6**	CDK6	Q00534	CHEMBL2508	**Kinase**
**Cyclin-dependent kinase 4**	CDK4	P11802	CHEMBL331	**Kinase**
**Cyclin-dependent kinase 9**	CDK9	P50750	CHEMBL3116	**Kinase**
**Inhibitor of nuclear factor kappa B kinase beta subunit**	IKBKB	O14920	CHEMBL1991	**Kinase**
**Inhibitor of NF-kappa- B kinase (IKK)**	CHUK	O15111	CHEMBL3476	**Kinase**
**Signal transducer and activator of transcription**	STAT	P42226	CHEMBL5401	**Transcription factor**
**Serine/threonine- protein kinase mTOR**	**MTOR**	**P42345**	**CHEMBL2842**	**Kinase**

Supplementary table S4: Drug likeness and ADME property of GBM-N019

Physicochemical Properties	GBM_N019	Acceptable limit
**Molecular Weight**	413.92 g/mol	≤ 500
**Violation of Lipinski's rule**	0	0
**No of Rotatable bonds**	7	<10
**No of H-bond donor**	3	≤ 5
**No of H-acceptor**	4	≤10
**Molar refractivity**	118.41	40-130
**Total polar surface area**	102.49 Å^2	≤ 100
**Consensus Log Po/w**	4.02	≤ 5
**Synthetic accessibility**	3.26	1(best) - 10(poor)

Supplementary table S5: molecular docking profiles of GBM-0N19 with STAT3, mTOR and CDK6

	NSC777205_mTOR	DIS (Ă)	NSC777207_CDK6	DIS (Ă)		NSC777207_STAT3
ΔG=(Kcal/mol)	-7.6		-8.1			-6.9
No. hydrophobic contact	4		3			
Conventional H-bond	ASN1899<br>GLN1937	2.79<br>3.07	VAL64<br>VAL77	2.80<br>2.07		ASN5
C-H bond	PRO1940	3.59	HIS78	3.39		
Halogen bond						GLN66
π -sigma	GLN1937					
π -cation						
π -alkyl	ILE1939<br>LEU1900		ARG78			
π -π stacked						PHE33<br>TYR79
Amide-π stacked			GLU72			
Van der waal forces	GLN2200<br>ALA1971<br>LEU1939<br>ASP2145<br>PRO2146<br>GLY1897<br>ASN1898		PRO74<br>LYS160<br>PHE135<br>LEU135<br>LEU68<br>VAL76<br>LEU65<br>GLU69<br>LEU79			PHE71<br>GLN67<br>ARG70<br>PRO36<br>SER2<br>ALA35<br>GLN32

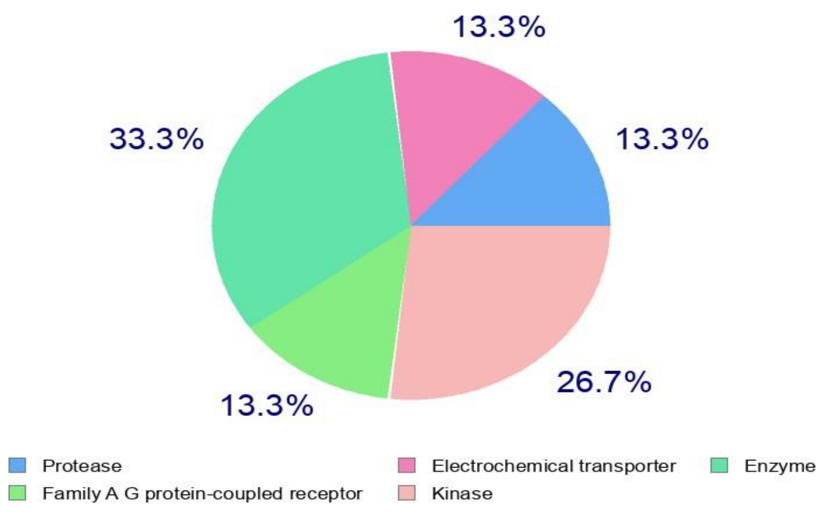

Supplementary figure S1. SWISSADME predicted classes of GBM-N019 potential drug target

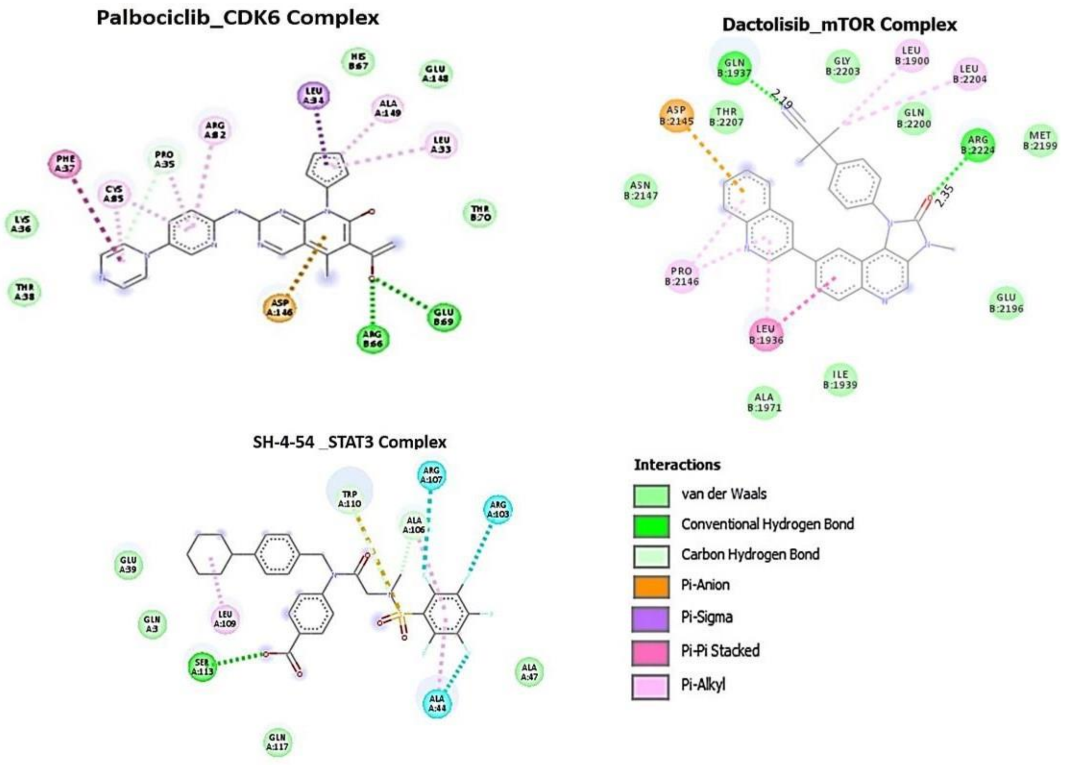

Supplementary Figure S2: molecular docking profiles of standard inhibitors with STAT3, mTORand CDK6

Figure 4E

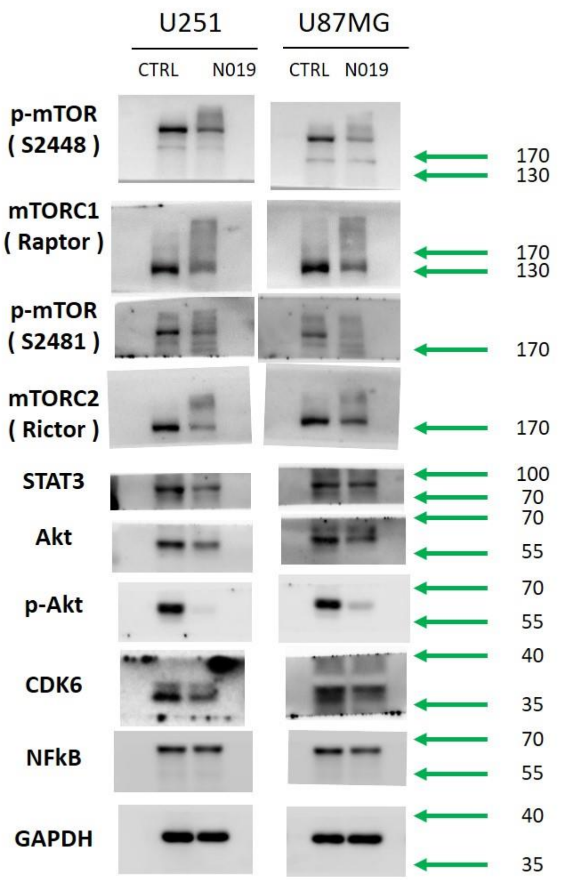

FIGURE 5C

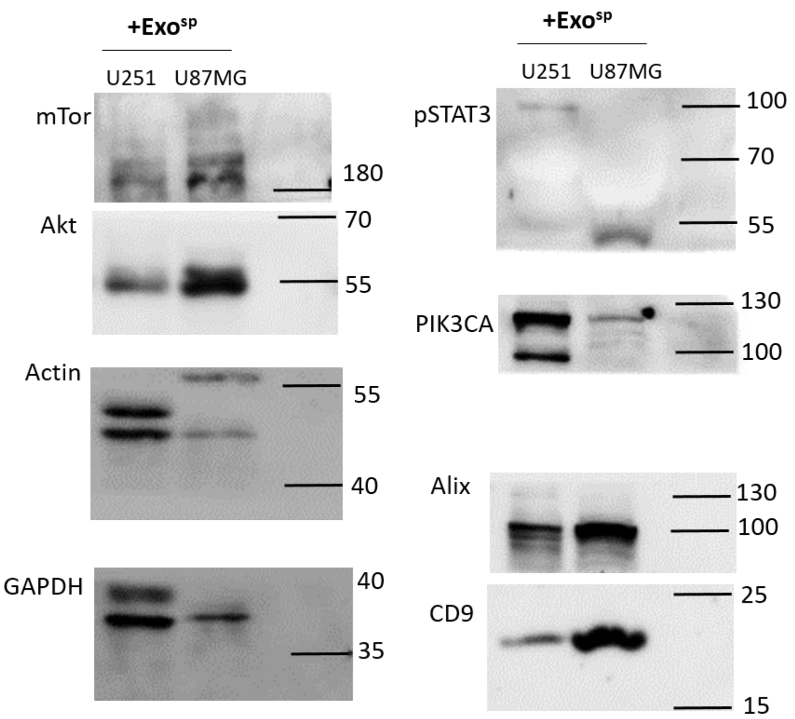

Figure 6D

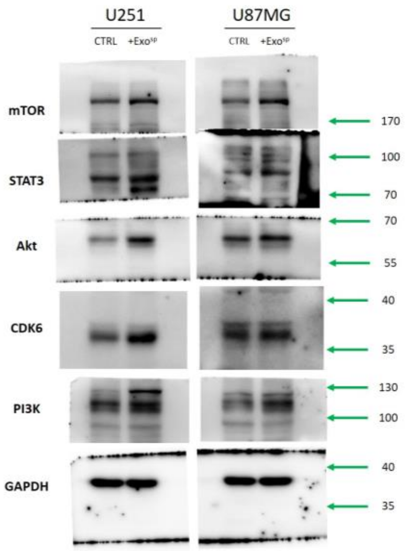

FIGURE 7D

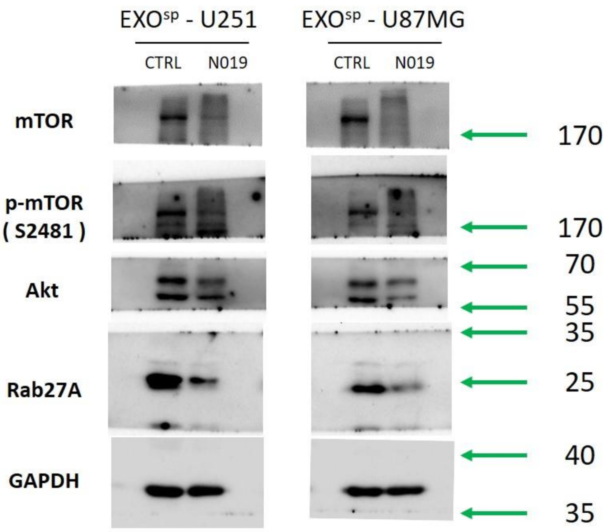

Figure 7. (F)

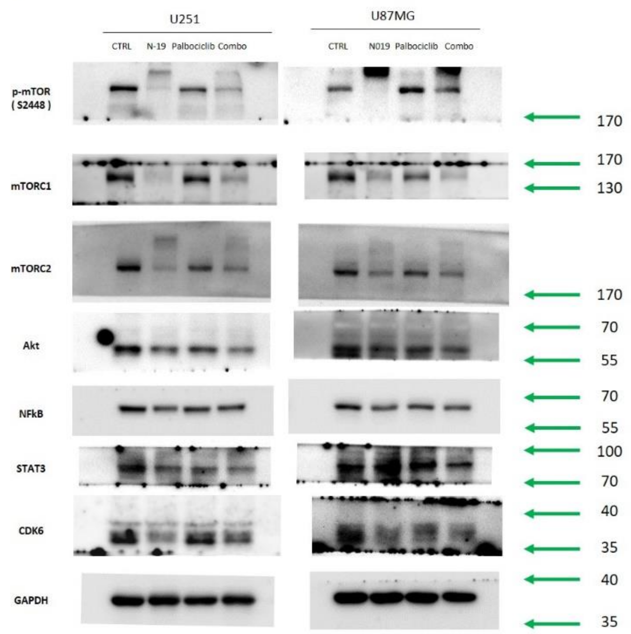

FIGURE 8D

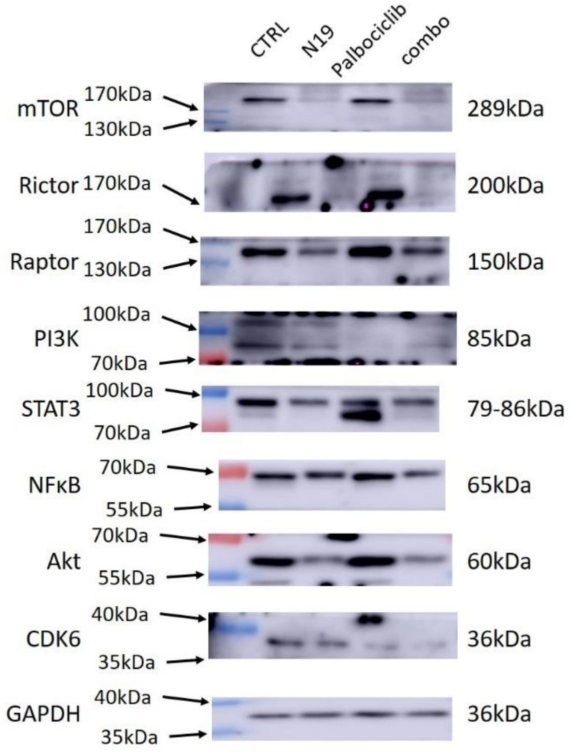

Supplementary Figure S3: Uncropped images of Western blots used in the figures
